# The dynamic interplay between anxiety-related, psychotic, and suicidal experiences: a qualitative study

**DOI:** 10.1186/s12888-025-07547-z

**Published:** 2025-11-19

**Authors:** Patricia Gooding, Kamelia Harris, Sarah Peters, Gillian Haddock

**Affiliations:** 1 Division of Psychology and Mental Health, School of Health Sciences, Manchester Academic Health Sciences Centre, University of Manchester, Manchester, UK; 2 Greater Manchester Mental Health NHS Foundation Trust, Manchester, UK

**Keywords:** Suicide, Anxiety, Non-affective psychosis, Schizophrenia, Hallucinations, Delusions, Paranoia

## Abstract

**Background:**

Suicidal experiences are highly prevalent in people with non-affective psychosis, as are anxiety problems. Understanding the interplay between suicidal and anxiety-related experiences in people with psychosis has been somewhat neglected. The over-arching aim of the current study was to redress this gap using a qualitative approach.

**Methods:**

A secondary Framework Analysis was applied to qualitative interviews with 18 people with recent suicidal and psychotic experiences.

**Results:**

Qualitative analyses evidenced a complex dynamic between psychotic, anxiety-related, and suicidal experiences. An emotional and cognitive-emotional dynamic was central which reflected: (a) the prominence of fear; (b) the perceived relentlessness of mental health problems; (c) feeling overpowered, overwhelmed, thwarted, and defeated; (d) perceptions of no hope and no future; and (e) wanting an unlikely end to the trap of ‘mental illness’. Within the emotional and cognitive-emotional dynamic, two pathways were clearly discernible: i.a. direct influence of anxiety on suicidal experiences with psychosis exacerbating anxiety-related experiences; and ii. an explicit pathway between psychotic and suicidal experiences with anxiety worsening psychotic experiences. A third pathway captured a non-discernible ‘mixture’ of anxiety-related, psychotic, and suicidal experiences. A fourth pathway illustrated how depressed and low mood states could interact with psychosis and anxiety to trigger and/or worsen suicidal states of mind.

**Implications:**

It is vital to better understand the interplay between psychosis, anxiety-related, and suicidal experiences whilst conjointly developing suicide-focused psychological therapies so that they directly address ways in which different manifestations of anxiety interact with psychosis to lead to, and worsen, suicidal experiences.

**Trial registration:**

ClinicalTrials.gov (NCT03114917), first submitted 29th March 2017 (29/03/2017), first submitted that met QC Criteria 10th April 2017, first posted 14th April 2017 (14/04/2017). ISRCTN (reference ISRCTN17776666 10.1186/ISRCTN17776666); 5th June 2017, (05/06/2017). Registration was recorded prior to participant recruitment commencing.

**Supplementary Information:**

The online version contains supplementary material available at 10.1186/s12888-025-07547-z.

## Introduction

Anxiety disorders are highly prevalent, occur across the lifespan, and when severe, can be exceptionally debilitating with negative consequences in numerous social, educational, recreational, and occupational domains of everyday life [1]. The impact of anxiety on suicidal thoughts and acts has been somewhat neglected. An early meta-analytic review which examined the relationships between anxiety disorders and suicidal ideation and behaviours presented evidence that suicidal thoughts, attempts, and deaths were more probable in individuals with a diagnosed anxiety disorder compared to those without an anxiety disorder [2]. Other reviews have emphasized a key role for a range of different presentations of anxiety in suicidal thoughts, plans, and acts. These anxiety presentations include Post-Traumatic Stress Disorder [3], Obsessive-Compulsive Disorder [4], Body-Dysmorphic Disorder [5], panic disorder [6, 7], and social anxiety disorder [8]. Furthermore, it is important to both acknowledge and better understand how the lived-experience of anxiety can vary markedly in severity, have a breadth and fluidity that may not easily fit with traditional diagnostic categorical boundaries, and overlap with other mental health problems, including affective disorders [9], and delusional and paranoid thoughts and feelings [10–12]. Indeed, as Skodlar and colleagues commented in the context of working with people with schizophrenia who had suicidal experiences “it is important for the clinician to be aware that behind the apparently common and familiar cliché complaints (such as “depression,” “anxiety,” etc.), there may hide more foundational disorders, which often resist description” [13], page 486]. Hence, in the current work our broad aim was to explore this breadth of anxiety-related experiences in relation to psychotic and suicidal experiences.

Non-affective psychosis, including schizophrenia, is characterized by the presence of hallucinations, delusions, and/or paranoid thoughts, feelings, and beliefs [14]. Rates of death by suicide are significantly raised in people with non-affective psychosis [15]. For example, a large linked database study in Canada found that 1 in 58 individuals with a schizophrenia spectrum diagnosis died by suicide within four years of that initial diagnosis [16]. In the UK, data collected between 2008 and 2021 from the National Confidential Inquiry into Suicide and Safety in Mental Health (NCISH) reported that 16% (2828/18,487) of individuals who died by suicide and were in contact with mental health services prior to death, had a diagnosis of schizophrenia, amounting to an average of 202 deaths per year [17]. Associations between hallucinations, including command hallucination, and suicidal thoughts, plans, and acts have also been reported across a range of clinical samples [18–21]. A general population survey documented a strong association between paranoia and suicidality [22]. Estimates of suicidal ideation, suicidal thoughts with intent to die, and suicide attempts in a sample with persecutory delusions were recently recorded as 23.6%, 11.8%, and 4.5% respectively [23]. It should be noted that whilst many individuals find the traditional psychiatric diagnostic medical model to non-affective psychosis helpful because it can provide both a framework that aids understanding and medications which offer a form of relief, some clinicians, researchers and service-users have questioned and challenged this approach [24, 25]. Challenges have emphasised the need to understand the complexities of environmental and developmental factors contributing to psychosis, in particular, the specific role that adverse events and trauma has been shown to play [25–30]. Perhaps, most importantly, the necessity of focusing on experiences of psychosis has been advocated [31–33]. People with psychotic experiences often have high levels of anxiety as evidenced by work with both clinical [10, 34–36] and non-clinical samples [11, 37]. The anxiety experienced may be akin to, for example, generalised anxiety, anxious anticipation or worry, panic, social anxiety and/or the resultant emotions of being in environments perceived as threatening [38–40]. Several cognitive-behavioural models of psychosis have emphasised the role of anxiety in the experience of psychosis [41–44], both as a response to manifestations of psychosis but also arising independently of psychotic experiences. For example, in a recent study comprising over 10,000 adults, structural equation modelling showed how anxiety and paranoia may share many common socio-cognitive explanatory pathways (e.g., avoidance; worry; negative beliefs) but can also be differentiated [12]. Similarly, experiencing hallucinations has been frequently associated with anxiety, with some evidence indicating a temporal relationship in which anxiety, anxious anticipation, and panic in particular, triggered and/or intensified hallucinations [40, 45].

Despite potential explanatory socio-cognitive mechanisms being identified between anxiety and psychotic experiences [12] and despite hallucinations, paranoia, and delusions being associated with high levels of suicidal thoughts and acts, there is a clear gap in the literature which is the need to better understand the ways in which anxiety-related experiences interact with psychosis to trigger and/or exacerbate suicidal mind-sets and behaviours. Transdiagnostic psychological models of suicide have highlighted the importance of thoughts and feelings that cluster around appraisals of being isolated, a burden, defeated, trapped, and hopeless [46–48]. Other work has highlighted how anomalous experiences of the self, such as ‘otherness’ and a fundamental sense of not belonging, impact feelings which go beyond isolation to depict an intense and unrelenting solitude, in people with psychotic and suicidal experiences [49–52]. The extent to which these types of thoughts and feelings remain central and become even more pervasive when non-affective psychosis, anxiety, and suicidal experiences interact is comparatively underexplored. Furthermore, although there is some evidence that variability in affect, confusion about emotions, and feeling unable to accept emotions [53–55], play an important role in pathways to suicidal experiences, the ways in which interactions between anxiety and psychosis affect the intensity and impact of emotional experiences is poorly understood, nor do we know how such emotions subsequently impact anxiety-psychosis-suicide dynamics.

These sorts of dynamics are often fluctuating, intersectional, and complex. This means that they are most optimally addressed with qualitative methods and analyses. Thus far, published qualitative work examining these kinds of dynamic appears somewhat sparse. In one exception, which used Interpretative Phenomenological Analysis (IPA) with one individual, fear arising from psychotic experiences particularly delusions, a perceived lack of control over anxiety, and the complex manner in which anxiety and delusions were intertwined, were identified as being particularly detrimental [56]. This type of work holds explanatory promise and should be expanded and built-upon.

Hence, the overarching goal of the current study was to understand how anxiety-related and psychotic experiences become interwoven to trigger and/or amplify suicidal experiences. The first aim was to explore how the interplay between anxiety, psychotic, and suicidal experiences gave rise to, and influenced, specific emotional and cognitive-emotional experiences, and how these emotional states then fed-back into the psychosis-anxiety-suicide dynamic. The second aim was to examine ways in which suicidal experiences emanated from, and/or became exacerbated by (i) anxiety experiences impacting psychotic experiences, (ii) experiences of psychosis impacting anxiety, and (iii) depressed mood states intersecting with anxiety and psychosis to affect suicidal experiences.

A qualitative methods approach was used, specifically Framework Analysis [57], with participants identified who had non-affective psychosis together with suicidal and anxiety-related experiences [58–60]. Framework Analysis was selected above a theory building approach, such as Grounded Theory, because the analyses were influenced by recent suicide models which place appraisals of emotional difficulties, negative social relationships, interpersonal problem solving difficulties, defeat, entrapment and hopelessness as central in pathways to suicidal experiences [46–48].

## Methods

### Settings

There were two settings: (i) The Cognitive AppRoaches to coMbatting Suicidality (CARMS) project [58, 60]: and (ii) The Resilience, Psychosis and Suicidality (RePS) project [59]. Both projects investigated inter-relationships between suicidal and psychotic experiences with qualitative and quantitative workstreams.

### Service-user involvement and engagement

Service-User Involvement and Engagement was embraced in two ways. First, two groups of Experts-By-Experience (EBEs) formed Service User Reference Groups (SURGs), one for CARMS and one for RePS. Second, one co-author is an EBE with anxiety and suicidal experiences. Members of the SURGs had all experienced severe mental health problems including anxiety, psychosis, and suicidal thoughts/acts. They advised on all stages of the research processes making particularly significant contributions to developing a glossary of anxiety-related terms^1^ and participating in a workshop led by KH about the ways in which anxiety can lead to, and escalate, suicidal experiences. The workshops were run during the refinement of the research questions and the development of the analytical framework.

### Participants

Participants were required to be aged 18 or over; have non-affective psychosis in accord with International Classification of Diseases 10th edition (ICD-10) criteria; have self-reported suicidal experiences (e.g., thoughts, feelings, urges, images, plans, and/or attempts) in the three months prior to recruitment (CARMS) or during their lifetime (RePS); not to require an interpreter to complete assessments and interviews; be under the care of a National Health Service (UK) mental health team with an assigned care co-ordinator; and be able to give informed consent as stipulated by ethical research governance procedures. Exclusion criteria were dementia or organic brain disorder; and currently participating in a psychological intervention clinical trial (CARMS study only). Particpants have been identified throughout with pseudonyms.

### Data generation

One-to-one qualitative interviews were carried out at one time-point and were transcribed, and analysed as part of the qualitative work-streams for the CARMS trial [58]. The topic guides explored (i) psychological pathways underpinning psychosis and suicidal experiences [61]; (ii) acceptability and feasibility of suicide-focused psychological therapy [62]; (iii) ways of optimising the implementation of a suicide-focused psychological therapy [63]; and (iv) the experience of being part of suicide research [64]. The RePS interviews were conducted by KH with topic guides asking about (i) the pathways underlying psychological resilience to suicide when living with psychosis, and (ii) transitions from suicidal thoughts to acts, and counters to those transitions [59]. It should be noted that the topic guides did not explicitly focus on anxiety, meaning that any anxiety-related experiences were spontaneously generated by participants. Hence, the current work was based on secondary qualitative data analysis.

Transcripts were purposively selected from the CARMS and RePS data corpuses so that we only included participants who had anxiety-related experiences in addition to suicidal and psychotic experiences. This was done in two steps. First, automatic text searching (using NVivo) along with analyst searching was applied to each potential interview for descriptors and labels indicative of anxiety-related experiences. To aid the search processes, a glossary of anxiety-related descriptors was developed based on (i) the extant literature including relevant peer-reviewed publications, reports (e.g., Samaritans), and websites (e.g., MIND, Anxiety UK), (ii) SURG member input, and (iii) the interview transcripts. The glossary was reviewed and expanded iteratively. Second, transcripts were read with an initial focus on identifying any exposition about (i) anxiety-related experiences, and (ii) the interplay between anxiety, suicide, and psychotic experiences. Transcripts were included if they appeared to contain sufficient material to address the aims.

### Ontological position and reflexivity

A critical realist perspective was adopted, whereby it was assumed that participants’ accounts reflected their subjective reality, co-constructed by the interviewer and interviewee in the interview process, but that there are also influences on any perception which manifest regardless of awareness of those influences [65, 66]. We used both a manifest and a latent level of analysis to collate and integrate the patterns of data identified. The research team comprised individuals with diverse experiences and perspectives including lived-experience of both anxiety-related and suicidal experiences, long-standing experiences of conducting highly sensitive qualitative work in numerous clinical and health psychology settings using a range of methods and techniques, extensive clinical work with people with non-affective psychosis and other severe mental health problems, and over two decades of clinical-scientific experiences in attempting to advance the psychological understanding of suicide and resilience to suicide in tandem with developing suicide-focused psychological interventions. During each stage of data generation and analysis, including team discussions, researchers reflected explicitly on their personal, scientific, and clinical perspectives that they brought to the process. Interviewers kept journals during the data generation phase, and the lead analyst (PG) kept a reflexive log detailing how their initial perspectives were altered and/or challenged as analyses progressed.

### Analytical procedures using framework analysis

Framework analysis takes a flexible, pragmatic, epistemological approach which can embrace both deductive and inductive components of qualitative data analyses [57, 67, 68]. Framework analysis has two phases of (i) developing an analytic framework, and (ii) applying the framework to the data. Seven stages were followed of [1] becoming familiar with the data; [2] identifying a transcript as the Framework Primary Transcript; [3] developing an agreed framework matrix; [4] producing a comprehensive summary for each individual transcript; [5] extracting and indexing data against the framework matrix; [6] a charting process to identify, clarify, and summarize relationships and patterns within the indexed data; and [7] mapping and interpretation of patterns found within the framework and the summaries [69].

#### The framework primary transcript

During the initial familiarisation phase of the analysis, one participant’s transcript Alex was identified as the Framework Primary Transcript (FPT) because they described ways in which anxiety and psychosis interacted to trigger and exacerbate suicidal experiences at considerable length, with anxiety being a prominent precursor of suicidal thoughts, plans, and attempts. This person’s experiences formed the basis for the development of the framework and the structure of the summaries. The summaries which were developed for each participant documented (i) their history of mental health problems; (ii) pathways linking psychotic, anxiety-related, and suicidal experiences; and (iii) the emotional and cognitive-emotional experiences relating to anxiety, suicide, and psychosis. Table 1 presents examples of two summaries, but in a less extensive format than used in the analysis to protect against participant identification. The fifth category reflecting the Influence of memories on the interplay between psychotic, suicidal, and anxiety-related experiences also arose from participant interviews and depicted ways that memories of, for example, previous anxiety episodes, panic attacks or past abuse affected current experiences.

**Table 1:** Examples of summaries for two participants in a truncated format to protect against participant identification. Summaries included history of mental health problems and mechanisms underpinning pathways involving psychosis, anxiety and suicidal experiences. One participant, Alex], experienced paranoia, and the other, Bo, experienced hallucinations. The transcript for ID008 was the framework primary Transcript. The symbol ‘=>’ represents Temporal precedence

ID	Alex
History	This participant had been experiencing psychosis, including paranoia, feeling persecuted, and anxiety in the form of extensive, often daily, anxiety or panic attacks, together with suicidal thoughts, plans, and/or attempts for over ten years. They had attempted suicide three times (overdoses and hanging) They made explicit suicide plans, and they took no action to abort their attempts. They had been detained under the Mental Health Act on psychiatric in-patient wards for extensive periods.
Brief mechanisms	Paranoia and delusions = > anxiety attacks/panic attacks + physical anxiety experiences = > suicidal thoughts, plans and attempts
Mechanisms explained	The pathway to suicide was clear for this participant, in that anxiety led to suicidal thoughts, plans and attempts. A precipitant and amplifier of anxiety was identified by this individual as paranoia and delusions. For example, hearing people outside talking was interpreted by the participant as meaning that people were talking about them. A complex anxiety dynamic built up over many hours, starting with disconcerting, almost alien, bodily physical sensations, accompanied by intense fear, and spiralling negative thoughts about the unknowns of these experiences and if, or when, they would abate, or conversely, if and how they would become worse. As these sensations, emotions, and thoughts amplified in intensity and duration, the participant started to feel suicidal (see indicative quotes 1 and 2). This complex dynamic was exceptionally frightening and overwhelming, compounded by not understanding what was happening to them.
Emotional and Cognitive-Emotional experiences	This participant experienced immense fear which was because of the intensity and the duration of the anxiety, compounded by not knowing if the intensity was going to get even worse, and not knowing, when, or if, the anxiety would abate. Consequently, anxiety felt overwhelming. Not knowing when the anxiety was going to end, and feeling unable to control it amplified the fear. The participant was desperate for the anxiety to stop but that did not seem to be happening for them in the here-and-now. Remembering what previous anxiety episodes had been like just escalated the need for the anxiety to stop, which was harshly juxtaposed with beliefs and fears that it would not stop.
Indicative quotes	1) *“I just want it to stop sorta…If it happens again like*,* I might do something*,* or something like that…Like suicide or something if it doesn’t go away*,* so… ‘cause it lasts such a long time*,* it’s just… like ahhhh…”* 2) *“Participant: In the [anxiety] episode*,* I hang myself and overdoses.* *Interviewer: Is that what actually happened because you didn’t know when it was going to end*?*Participant: Yeah*,* yeah.”*
ID	**Bo**
History	This participant had attempted suicide twice, once by hanging and once by overdosing with medication. They felt suicidal a few months prior to interview and they reported feeling suicidal daily. This person had multi-modal hallucinations which also occurred daily. They self-harmed and attempted suicide because of commands from the voices. They experienced both worry and agitation often because of threats by the voices to harm someone close to them and because the voices seemed omnipotent which was related to their self-harm and suicidal thoughts and attempts (see indicative quotes below). This individual also described a more general type of anxiety that happened before trying to carry out daily activities.
Brief mechanisms	Two routes: (1) Anxiety, agitation, stress, worry, panic + voices = > suicidal thoughts and attempts; (2) Stigma = > other people being denigrating = > suicidal thoughts/feelings.
Mechanisms explained	Voices threatened to harm someone who the participant was close to if the participant didn’t hurt themselves and/or kill themselves, and the participant believed that this could happen. This participant felt very anxious and agitated in parallel with hearing the voices. In addition, voices made this person feel exceptionally worried (as described by the participant – see quote 3 below). Added to this was the fear of not knowing where the voices were coming from (i.e., it was an unknown). This person also described a direct link between people putting them down because of their psychosis and suicidal thoughts and feelings.
Emotional and Cognitive-Emotional experiences	This participant felt that their voices were overpowering and unrelenting (they were there every day), exacerbated by not knowing where the voices were coming from. When the voices commanded this person to hurt themselves and to kill themselves, they felt as though the voices were taking over. They also described feeling very scared of the voices and images. It was the realness of their voices and images which was compelling and frightening. This participant also described feeling embarrassed about the stigma towards them because of schizophrenia.
Indicative quotes	1) *“Yeah*,* I do* [experience voices], *when I get anxious as well.”* 2) *“But I get very anxious before I do things.”* 3) *“And when I get worried about [person] too much*,* I sometimes*,* I try to hang myself as well.”* 4) *“the voices are taking over and I don’t want [person] to get hurt*,* they say they’ll stop hurting [person] if I kill myself.”* 5) *“It’s all… they* [voices] *feel so real.“*

The final framework had five sections which documented suicidal, psychotic, and anxiety-related experiences and included categories derived both deductively and inductively (see Table 2). For example, a focus on perceptions of hopelessness, defeat and feeling trapped in the Emotional and Cognitive-Emotional experiences category was based on robust published evidence that these appraisals are strong predictors of suicidal thoughts/behaviours [70–72]. In contrast, (i) emotional experiences, (ii) disrupted thoughts, and (iii) perceptions of having no control, being over-powered, and the relentlessness of mental health problems within this category originated from participant interview data. The fifth category reflecting the Influence of memories on the interplay between psychotic, suicidal, and anxiety-related experiences also arose from participant interviews and depicted ways that memories of, for example, previous anxiety episodes, panic attacks or past abuse affected current experiences.

**Table 2:** The framework matrix used as part of the qualitative approach to analysing the interview transcripts organised in five sections

1. History of, and current, suicidal experiences (e.g., feelings, thoughts, images, plans, attempts)
2. Characteristics of anxiety manifestations
a. Anxiety diagnostic ‘types’ (e.g., PTSD, panic attack, social anxiety, phobias) plus duration, frequency and intensity
b. Physical feelings (e.g., restlessness in limbs, shakiness, agitation, spasms, cramps)
c. Behaviours (e.g., avoidance, purging)
3. Psychotic experiences as expressed by participants (e.g., hallucinations, delusions, grandiosity, dissociations, psychiatric diagnostic labels)
4. Emotional and Cognitive-Emotional experiences of the interactions between psychosis, suicide and anxiety
a. Emotions (e.g., fear, anger, frustration, resentment, irritability)
b. Disrupted thoughts (e.g., confusion, racing mind, spiralling thoughts, rumination, preoccupation, worry, hypervigilance)
c. Perceptions of hopelessness, defeat, being trapped
d. Perceptions of being controlled and/or being overpowered, feeling overwhelmed, losing control, and/or the relentless of mental health problems
e. Other specific thoughts and/or cognitions (e.g., “I’m in a dark room”)
5. Influence of memories on the interplay between suicide, psychosis, and anxiety (e.g., memories of previous anxiety attacks; sexual abuse)

The framework and the summaries were integrated, with the summaries acting as an anchor point for the mapping and interpretation processes (stage 7). This integration process allowed patterns to be identified and interpreted across transcripts in which anxiety, psychosis, and suicidal experiences were intertwined. At the outset, analysts were mindful of different manifestations and characteristics of anxiety-psychosis-suicide interactions and dynamics, for example, fluctuating, cyclical, bidirectional and non-linear inter-relationships. It should be noted that instances where it seemed clear that anxiety, psychosis, and suicidal experiences were mutually-influential but no discernible ‘patterns’ were evident were documented in their own right.

#### Procedure

From the initial stages of the analysis process, it was clear that anxiety-related experiences were highly diverse. Hence, a glossary of over 90 inductively and deductively generated anxiety descriptors was used to help analyst-led identification of anxiety-related information in the transcripts (e.g., tetchy, “body is squeezing itself”, terror, restlessness, overthinking).^1^ This formed a starting point. Initially, PG and KH developed the framework and structure for the summaries by independently coding four transcripts iteratively with refinement applied at each iteration. Thereafter, PG applied the framework and summary structure to the transcripts. The final interpretations of the data patterns across the transcripts, and the explanatory narrative were discussed and fine-tuned with input from the wider research team to agree the final choice of illustrative quotes and the conceptual model. PG was the primary and lead analyst. KH was a second analyst. As a highly experienced qualitative methodologist and health psychologist SP supervised all stages of the analysis. GH provided interpretative input from the perspectives of a clinical psychologist specialising in psychosis. Qualitative analyses processes were organised and collated with NVivo software (version 12.0, QSR International Pty Ltd, 2018) including the reflexive logs of analysts together with Excel.

## Results

### Participant characteristics

Eighteen interview transcripts were included in the qualitative analyses (mean length = 54.6 min, SD = 25.7 min). The median age of the 18 participants was 38.5 [21–64], with 15 being male (3 female), 16 being white with 1 identifying as black, and 1 of mixed race. Fifteen participants were single, 2 were in a relationship, 1 had missing data. Seven participants were unemployed, 4 were exempted from employment, 4 did volunteering work, 1 identified as a housewife, 1 was a student, and 1 was employed. Ten interviewees lived alone, 5 lived with parents, and 3 lived in supported accommodation. Nine participants had a diagnosis of schizophrenia, 5 had paranoid schizophrenia, 3 had a diagnosis of schizoaffective disorder, and 1 had a diagnosis of psychotic disorder and depression. Examples of suicidal and psychotic experiences as relayed by participants in their own words have been summarised in supplemental Table S1.

### The interplay between suicidal, psychotic, and anxiety-related experiences: overview

**Fig. 1:**
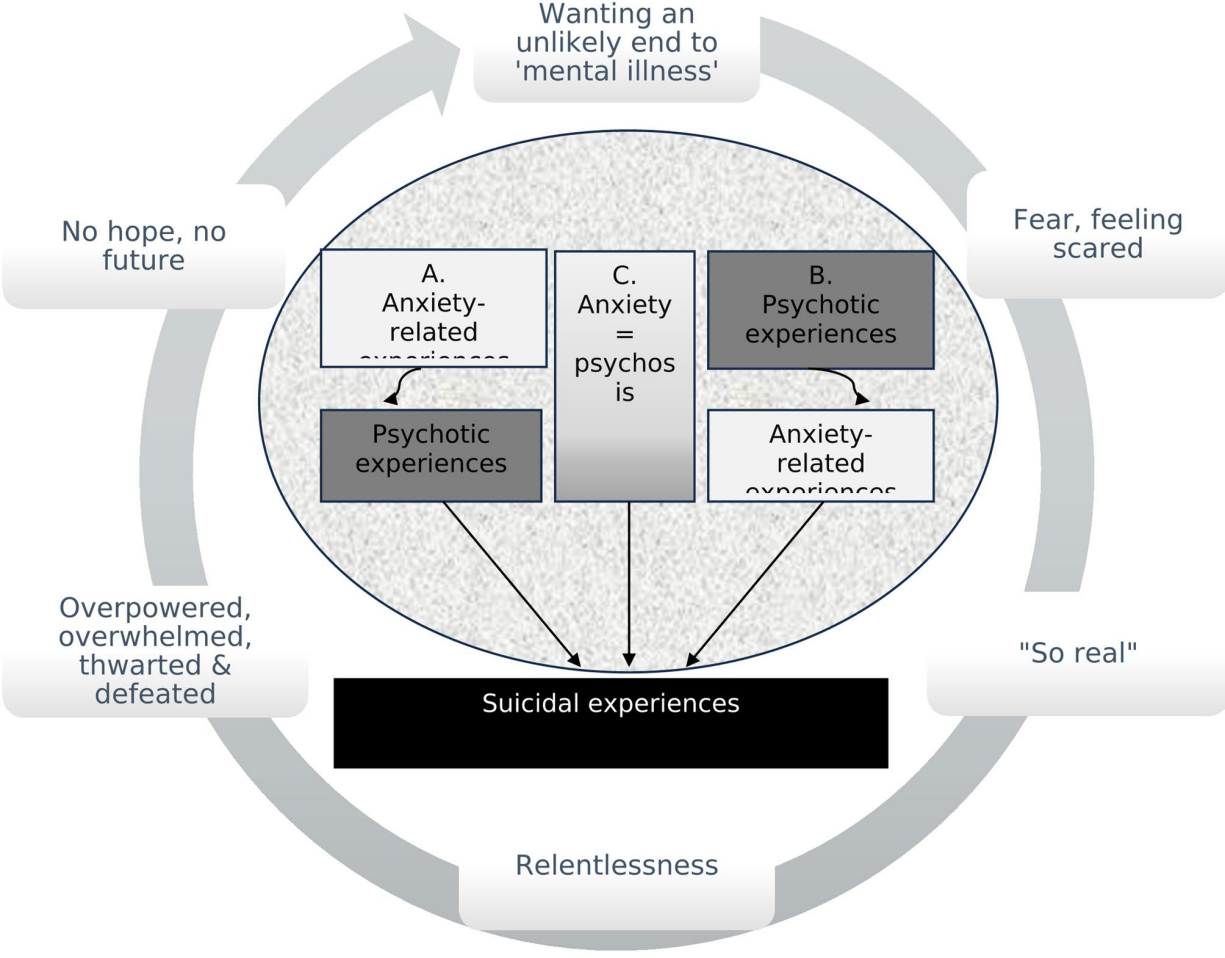
Conceptual diagram of the dynamic interplay between anxiety-related, psychotic, and suicidal experiences which was relayed by participants. Three pathways (**A**, **B**, **C**) to suicidal experiences are depicted which are situated within 6 over-arching cognitive-emotional concepts of (i) fear and feeling scared, (ii) psychotic experiences feeling ‘so real’, (iii) the relentlessness of psychotic and anxiety-related experiences, (iv) feeling overpowered, overwhelmed, thwarted and defeated, (v) having no hope and no future, and consequently (vi) wanting an unlikely end to ‘mental illness’. The first pathway **A**, represents participant accounts in which anxiety-related experiences seemed to precede and/or trigger psychotic experiences, and these psychotic experiences, in turn, fed-into suicidal experiences. In pathway **B**, psychotic experiences fed-into anxiety which then impacted suicidal thoughts and acts. In pathway **C**, psychotic and anxiety-related experiences felt indistinguishable to participants. For some individuals, feelings of depression and low mood also fed into each of these pathways (represented by the speckled oval). It should be noted that even though pathways contained elements of temporal precedence, they also had dynamic and fluid properties

The interplay between suicidal, psychotic, and anxiety-related experiences represented a complex dynamic. Central to this dynamic was an emotional and cognitive-emotional interplay which encompassed, triggered, and drove, pathways linking psychotic, anxiety-related, and suicidal experiences, some of which were impacted by depression and low mood. Figure 1 illustrates the emotional and cognitive-emotional interplay into which was woven the psychosis-anxiety-suicide pathways.

### An emotional and cognitive-emotional dynamic

Many participants referred to specific emotions accompanying anxiety-related, psychotic and suicidal experiences, for example, anger, frustration, feeling upset, alone, lonely, resentment, “gloom and doom”, sadness, unhappiness, feeling awful, and a state of misery:

Interviewer: *And so in terms of feeling like you don’t want to stay alive*,* I know we’ve sort of spoken about it in little bits*,* and you said*,* you say you feel like you have more reasons now*,* but you still get those kind of daily thoughts. And like what’s that like*,* how is that to live with?*

Participant: *Miserable. For lack of a better word it is absolutely miserable. But it is*,* and I survive [Bobbie]*

However, for others, emotional descriptors were either hard to pin-point *(“I can’t describe the feeling*,* it’s horrible…” [Stevie]; “If it gets bad” [Kit]*) or were simply not adequate to capture their feelings. One participant communicated how medication could act to numb or suppress emotions:

*I spent a lot of years on Olanzapine… One of the reasons I don’t like it is it suppresses all your emotions*,* and I spent a lot of years on Olanzapine*,* surviving. I came off Olanzapine and I’d got a good insight into being well and being ill and I can recognise things… [Charlie]*

Another participant described finding it difficult to recognise their emotions and communicate about them, but found that this was helped by writing poetry:

*I started writing poetry about how I felt*,* and it was really dark*,* you know*,* really depressive*,* angry and what have you. And on a [day of the week]*,* we used to go to the pub and I got the confidence to read out some of my poetry… and the poetry got darker*,* and more suicidal*,* and angry … [Georgie]*

Across the interviews, an emotional and cognitive-emotional dynamic became apparent in which the pathways interlinking suicidal, psychotic, and anxiety experiences were embedded. Six constructs were identified which could provoke, drive, and exacerbate the psychosis-anxiety-suicide interplay. One of the most striking specific emotions that seemed common to many interviewees, and which comprised the first construct, was (i) Fear and feeling scared. Emanating from, and central to, these different manifestations of fear was the resultant perception that psychotic experiences were (ii) ‘So real’ which represented the second construct. Three cognitive-emotional experiences followed comprising three further constructs, namely, (iii) Being overpowered, overwhelmed, thwarted, and defeated; (iv) No hope and no future, (v) Wanting an unlikely end to ‘mental illness’, and vi Relentlessness. This sixth construct was present across these five emotional and cognitive-emotional constructs with such strength that it seemed fitting that it formed an identified construct in and of itself.

#### Fear and feeling scared

Fear and feeling scared were expressed by participants in relation to anxiety, hallucinations, delusions and paranoia. Fear could be associated with anxiety directly, *“it’s [anxiety attacks] scary and suicidal” [Alex]*; the hallucinatory voices themselves, *“I did it [self-harm and suicide attempts] quite a lot because it was scary seeing things in my head and that” Kit];* the negative self-deprecating content of hallucinations; images and/or voices of past abusers; not knowing where voices were coming from; the threatened actions of hallucinatory voices against participants or against other people if the participant did not comply with those voices; and fear of hurting other people as a consequence of delusional thoughts, feelings and beliefs. Perceptions of being taken over, losing control, or being erased by hallucinations and delusions provoked considerable fear for some participants, “*I can’t think for myself anymore. There’s some other entity that’s come in and taken over” [Jac]*. One participant described how they were afraid of going to sleep, *“Yeah. But when it gets too bad and it’s not happening for me and I can’t get any more rational thoughts*,* I mean this time*,* I was so scared of going to sleep. Don’t ask me why*,* but I’m so scared of going to sleep…” [Charlie]* which seemed associated with a dread of thoughts building-up and racing in ways that were far removed from making sense, *“like wires at the back of my forehead” [Charlie]*.

Psychotic and anxiety-related experiences failing to make sense was a central fear-inducing factor for a number of people we interviewed. This seemed most prominent for people whose anxiety involved intense physical sensations especially when those sensations seemed to move throughout their body, or for one individual when their anxiety produced spasms. Related to this was the impact of unknowns. With respect to anxiety, those unknowns encapsulated how long a panic attack or anxiety episode would last, whether the anxiety episode or panic attack would actually stop, and feeling unsure about how intense the anxiety would or could get, and relatedly, the extent to which the participant felt able to handle that intensity. The duration of anxiety episodes and panic attacks could be extensive, for example, over eight hours. Rationalisations that the participant had come through previous intense and prolonged anxiety episodes often failed to help because the extent to which the current anxiety episode would be worse in potentially multiple ways was an unknown threat for the participant.

#### ‘So real’

One striking and poignant aspect of fear was that even though many participants had insight into their psychotic experiences, those experiences still felt exceptionally real. It was not the anticipation of humiliating, challenging, or potentially traumatising events or situations that precipitated fear, but the ‘realness’, *“It’s all… they feel so real” [Bo]*, of both paranoid feelings/thoughts/beliefs and hallucinations. This was especially so when psychotic experiences indicated bodily harm (e.g., one participant felt that their arm was on fire); when they implied a disaster involving others such as failing to protect close family from an invasion or from being tortured; when they were linked to past trauma and abuse; or manifested as threats of harm to family to whom participants felt particularly close.

#### Being overpowered, overwhelmed, thwarted, and defeated

Feelings and perceptions of being overpowered, overwhelmed, and defeated by mental health problems were relayed across a number of interviews. Perceptions of being overpowered by voices which were highly invasive were often described:

*but if you can imagine taking your brain out and putting a little person and then they shout. I’ve experienced that. I don’t like that ‘cause that’s very invasive*,* I don’t know*,* I’ve got no control over it. [Georgie]*

This sense of being overpowered could feel unbearable especially when people were hearing more than one voice and when they built up in frequency and/or intensity:

*they’ll [voices] progressively through the day*,* they’ll get louder and more frequent*,* and louder and frequent*,* and louder and frequent until they get to the stage where at the night time I can’t handle it anymore and I end up taking handfuls of pills to like take away the voices… [Jude]*

This intensity and sense of being overpowered could become further compounded when intermixed with different kinds of anxiety. For example, anxieties concerning going out, worry about voices interfering with social conversations, anxiety about being watched which voices could in turn make worse, and anxieties about how and/or why the anxiety was occurring. Many participants also experienced agitation and restlessness which could make them stressed and then, in some cases, make both their voices and paranoid thoughts and feelings seem more negative, intense and/or frequent.

For some participants, defeat was prominent, *“Life’s defeated me that’s what it is. Yeah. Right. I’ve been defeated. So*,* I’ve lost*,* lost. I feel lost” [Jac].* Defeat was represented by not being able to fight anymore, *“I just give up fighting… Sometimes I felt defeated in the past. I mean*,* I felt like the voices had defeated me at times” [Kit];* feeling subjugated, *“I feel beaten down” [Jude*]; and sinking to the very bottom together with concomitant feelings of being worthless, *“I feel useless*,* like I’ve achieved nothing” [Kit].* These sorts of perceptions of being a failure could be reinforced by voices, *“They [voices] just talk to me*,* telling me I’m a failure” [Kit].* A slightly different perspective was also explicated by participants which was a fundamental perception of being thwarted, of stopping and/or being stopped, of not being able to carry on. These sorts of thoughts and feelings of being thwarted, having to stop, or just stopping, seemed all encompassing, akin to being eradicated.

Feeling cognitively and emotionally overloaded and/or overwhelmed, having a “*fuzzy mind” [Lou]*, and having racing thoughts and/or being in ‘overdrive’ were common for many participants. The experience of one participant was particularly arresting because that feeling of being overwhelmed didn’t just apply to racing thoughts, confusion, and/or anxiety but could also be instrumental in wanting to die by suicide:

Participant: *Yeah*,* yeah*,* I feel sui*,* I just*,* the only thing*,* I felt like tha*,* I had this overwhelming feeling of wanting to die on this particular day. The only thing I could…*.

Interviewer: what caused it, do you know?

Participant: *I’ve no I*,* jus*,* just the fact that I had this illne*,* it’s just*,* e*,* e*,* e I was just ill. It*,* It*,* I was just ill and had been for a long time*,* it was gonna be a lot*,* as ill as that for a long time after*,* it’s…*.

Interviewer: you mean mental, your mental health problems?

*Participant: [Coughs]*,* yeah*,* yeah*,* mentally ill*,* this war that was going on inside of my head and everything [Morgan]*.

The overpowering and consuming nature of mental health problems; feeling unable to fight; and a perception of being thwarted even to the point of not feeling human, all contributed to perceptions of being defeated for many. These kinds of defeat became coalesced with not only losing hope but being in a situation where there was no hope.

#### No hope and no future

Participants described a juxtaposition of hopelessness and suicidal thoughts, feelings, and acts. Sometimes hopelessness was a result of a culmination of negative life stressors and negative life events in addition to mental health problems:

*I was going to get a one-way ticket to–*,* train ticket to [Location 1] to jump off a cliff that I knew*,* so I was hopeless*,* I had nowhere to live*,* I had no money*,* I had debt*,* I had no car*,* I had no job*,* my life was just a mess. I was living illegally in an old people’s estate*,* so at that point I was hopeless; [Georgie].*

For other individuals having no hope was more obviously linked with their specific mental health problems. For instance, one interviewee had attempted to take their life with prescribed medication a week before the interview. Anxiety was a precursor to voices and was accompanied by a build-up of racing thoughts which precipitated suicidal thoughts, “*before I know it I’ve got a million things going on in my head*,* and that’s when I start thinking of taking my life*” *[Jude]* with the relentlessness of their voices leading to hopelessness and suicidal thoughts and plans, *“Yeah*,* hopeless*,* feel hopeless every day” [Jude].* A different participant *[Ollie]* added a slightly different dimension to these sorts of feelings and perceptions in that they conveyed how, for them, it was not so much that they felt hopeless but that there was no future for them; the future had gone – it was absent:

*so it’s like the future*,* you can’t think of it*,* it’s not there*,* it’s like it is gone. It’s weird even though you can think of the past but obviously not the future [Ollie].*

One prominent aspect that became clear across interviews was that even when asked directly, not all participants felt hopeless. It seemed that perceptions of not being able to take anymore, and relentless and overwhelming defeat seemed a stronger precursor to suicide than hopelessness. Hence, a strong desire was expressed by numerous participants for their mental health problems to abate. It is, perhaps, unsurprising that having to live each day with persistent thoughts and feelings which were characterised by an immense struggle, feeling utterly defeated, having no choices left, and having a future that seemed like a void, led many participants to speak about their desperation for their mental health problems to stop. That their mental health problems would abate seemed for many highly improbable which was strongly linked to their suicidal experiences.

#### Wanting an unlikely end to the trap of ‘mental illness’

In the current corpus of interviews, feeling trapped was evinced as wanting severe mental health problems, and ‘mental illness’, to stop but that not seeming likely or possible. Hence, the trap. One participant who described having ideas of grandeur, persecution, irritability, and recurring suicidal thoughts, explained:

*The strongest time that I felt suicidal was this time round and it was very recent. I think it was just not wanting to die*,* just wanting my illness to end […] You just start thinking*,* I don’t want to be around*,* I want out of this*,* and I don’t see any way out but to take my own life [Charlie].*

Participants expressed that they wanted an end to their suffering and to stop having to struggle. Wanting an end to mental health problems applied to anxiety, for instance, panic attacks and social anxiety and/or fear of going out. One participant *[Ollie]* who told us that they had “paranoid schizophrenia” struggled with stress originating from childhood, anxiety about going out and/or speaking to people, and feeling watched. They relayed:

*Because of my illness and I struggle*,* on a bad day I will think to myself I could just take all my tablets then I won’t have to suffer” […] it [paranoia] really gets you down*,* you know*,* it makes you wonder like is it worth living? Because is it going to carry on? [Ollie].*

#### Relentlessness

A potent contributing factor to these five emotional and cognitive-emotional constructs was the relentlessness of mental health problems. The impact of voices often increased throughout the day which also meant that the cognitive-emotional repercussions of what the voices said was cumulative. Paranoia was, for some, a constant in their daily lives. Similarly, delusions and delusional beliefs could grow to become all-encompassing. The relentlessness of these psychotic experiences increased even more when combined with different kinds of anxiety, including agitation, restlessness, worry, and having a racing mind. Anxiety, itself, could feel unremitting and punishing, *“Yeah*,* it [anxiety episode] was really intense*,* happening like every day for a few hours like… cause it’s lasting a long time and it’s quite intense as well” [Alex].* Consequently, these mental health problems could feel unending and unrelenting, “*I just feel like there’s no release or relief or anything” [Remy]*.

### Pathways between anxiety-related, psychosis and suicidal experiences

Two discernible and specific pathways were evident within the emotional and cognitive-emotional interplay in which (i) anxiety was central to suicidal experiences, with anxiety being triggered and/or heightened by psychosis, and (ii) psychosis played a pivotal role in suicidal experiences, with psychosis seeming synonymous with anxiety, or being triggered by anxiety. In contrast, distinguishable pathways could seem impossible to discern for some participants with psychotic, anxiety-related, and suicidal experiences seeming inextricably intertwined and portrayed as “a mixture” which constituted a third pathway. A final, fourth, pathway was evident in which depressed and low mood states affected the psychosis-anxiety-suicide interplay.

#### Anxiety was central to suicidal experiences, but psychotic experiences could trigger and/or heighten anxiety

In this first pathway, the centrality of anxiety to suicidal experiences was paramount, “*Erm*,* the anxiety was happening every day and I just couldn’t deal with it anymore*,* so… tried to end my life sorta” Alex* with anxiety and suicidal experiences occurring in tandem or with suicidal thoughts, feelings and/or urges building up during an anxiety episode or panic attack, *“The suicidal thoughts happen with the anxiety attack” Alex.* However, what was equally as paramount across participants was the ways in which hallucinations, paranoia and delusions could contribute to, and magnify, that anxiety. Many participants explained how paranoia often emanated from hearing people outside talking, or seeing groups of people in conversation, which they readily interpreted as talking about them in negative, hostile, or deprecating ways. Participants relayed feeling preoccupied and “stuck” with both the paranoid feelings and resultant anxiety.

For a number of participants, anxiety was described in terms of stress, which often co-occurred with escalating tension, for example *“getting worked up” [Robin].* Hallucinations, most often in the form of voices, but sometimes as voices together with images, fed-into and worsened these kinds of anxiety states. One participant who experienced both hallucinatory voices and images explained, *“so it’s like a double scene*,* they speak. When I hear both and I see both*…” *[Rowan]* and went on to describe a kind of agitated anxiety that seemed to have a tangible physical presence as they were becoming suicidal:

*But it wasn’t much feeling*,* it was*,* [short pause]*,* like physical feel–*,* I felt very agitated*,* very warm…So*,* but if I was going suicidal*,* I get like a tummy feeling*,* like*,* erm–*,* like if you miss a step on a stair*,* trip up*,* that kind of tummy thing… That’s what it felt like [Rowan]*

Specific characteristics of anxiety episodes worsened suicidal experiences, for instance, their frequency which could be a number of times a day over consecutive days or every day; the build-up in severity of the anxiety episode together with increasing intensity; the associated fear that the intensity would get worse; for some, the long duration and related expectation that the anxiety episode would not abate; and not understanding why the anxiety was manifesting, and manifesting in certain ways, for example, as muscle spasms. Suicidal thoughts arose from wanting these types of anxiety-related experiences to stop:

*I just want it [anxiety attacks] to stop sorta… If it happens again like*,* I might do something*,* or something like that…like suicide or something if it doesn’t go away*,* so… ‘cause it lasts such a long time*,* it’s just… like ahhhh… Alex]*

These presentations of anxiety in which psychotic experiences triggered, fed into, or worsening anxiety seemed to precede and provoke suicidal thoughts, feelings and urges. Wanting the anxiety to abate, but not feeling able to enact that level of imperviousness, combined with not understanding why or how anxiety was occurring in the ways that it was occurring, was instrumental in the development of suicidal feelings, thoughts and acts.

#### Psychosis played a pivotal role in suicidal experiences, with psychosis seeming synonymous with anxiety or being triggered and/or amplified by anxiety

In this pathway, psychotic experiences seemed most proximal to suicidal states of mind and suicidal behaviours, with different indicators of anxiety acting as a precursor to, or a magnifier of psychotic experiences. For example, one participant who experienced both very realistic hallucinatory images and voices, described how the voices threatened to hurt a family member unless they severely hurt themselves. Similarly, the voices bargained with this individual that if they killed themselves then they would not harm their relative. Hence, there was a direct link for this individual between their voices and suicide.

Interviewer: … so what is it that makes you feel suicidal and makes you want to take your life?

*Participant: … because of the voice*,* the voices are taking over and I don’t want [close relative] to get hurt*,* they say they’ll stop hurting [close relative] if I kill myself [Bo]*

For this person, anxiety triggered their voices and made them worse “*Yeah*,* I do [experience voices] when I get anxious as well” [BO]*. Furthermore, they worried almost constantly about their relative as a consequence of the threats from the voices, and became very agitated, with the worry feeding into suicide as a way of preventing their relative from being hurt. Although this participant’s situation was complex, a clear mechanism seemed to be operating in which anxiety either preceded or emerged simultaneously with their voices, and the voices resulted in them attempting suicide in order to appease those voices, with worry further amplifying their suicidal thoughts and feelings. A similar experience was relayed by a different participant who regularly heard a number of negative and derogatory voices, with anxiety and a racing mind feeding into the voices. This person had also been receiving messages from the television and sometimes from music, which interacted with the voices to bring about a suicide attempt:

*I was hearing voices at the time as well*,* you know*,* a combination of the voice and the TV got to me. So*,* I ended up taking an overdose then and I actually ended up in A&E [Sam]*

Clear links were also expressed by participants between delusional and paranoid thoughts, feelings, and beliefs, and suicidal experiences, with anxiety feeding into paranoia. For instance, one participant who told the interviewer that they found it difficult to find the words to describe their experiences, nevertheless communicated ways in which anxiety, manifesting as panic attacks and fear of those attacks, *“is that person going to be staring at me? Am I going to have a panic attack?“ [Stevie]*, activated and heightened their delusional and paranoid thinking, which led to, and was associated with, feeling unable to cope, self-harm, and suicide attempts.

#### A mixture: Anxiety, psychosis and suicide as a welded dynamic

Although the dynamics involved in the first two pathways were complex, they were still discernible. In contrast, psychotic, anxiety-related, and suicidal experiences were expressed by some participants as a highly inter-connected dynamic; a mixture:

*I can’t explain how I feel sometimes*,* it’s a mixture… Yeah*,* it is like a vicious circle*,* it all connects. So*,* if one experience happens it will lead onto the major experiences when I take my own life… [Ollie]*

One participant, who had been hearing different, exceptionally negative, and menacing hallucinatory voices since being a child that got progressively louder, more frequent, and more relentless throughout the day, relayed feeling suicidal a few times a week, and described co-occurring anxiety in the form of an intensely racing mind together with physical bodily sensations. They took prescription medication with clear intent to end their life. For this participant, their mental health problems and suicidal feelings seemed to merge into one entity:

Interviewer: Do you think there’s a link between the underlying problems you have and feeling suicidal, do you see it as something separate?

*Participant: No*,* I take them together me*,* I see it all as one thing. Yeah (Jude)*

Similar to other participants, the physicality of this participant’s anxiety was striking, as was their description of their racing mind which could become exceptionally intense. These sorts of experiences of a racing mind, manifesting as a kind of pressure, was common across a number of participants. Having a racing mind, and thoughts which built up in this way and couldn’t be disentangled, led to suicidal thoughts and acts which was part of escaping that escalating pressure.

#### The influence of depressed and low mood States on the psychosis, anxiety, suicide interplay

Depression and low mood were sometimes described by participants as impacting the interplay between psychotic, anxiety-related, and suicidal experiences. For example, one participant had attempted suicide around four months prior to the interview. They experienced hallucinatory voices, *“the voice would convince me that I was like*,* dead*,* I was a ghost” [Terrie]*, paranoia, and ‘strange thoughts*”* which developed when they were young adults. Anxiety, for them, was linked to being around groups of people and a feeling that other people were looking at them. Those sorts of paranoid thoughts and feelings resulted in them feeling low, useless, and drained of energy which, in turn, led to them having suicidal thoughts, possibly aggravated by wanting to disconnect from other people. So, this person’s experiences resonated with pathway ii. in that socially-related anxiety was one precipitant of paranoia, but that then led to suicidal thoughts via a depressed state:

*Erm*,* [long pause] I think the psychosis is what caused my depression and there’s like a direct link there and like*,* I’ve always been anxious around people [long pause]--*,* so*,* I don’t think that that’s too much of a problem*,* but like*,* the anxiety can trigger like*,* the symptoms of the psychosis.*

Interviewer: Okay. That makes sense, and what kind of things might trigger your symptoms?

*Participant: It’s groups*,* groups of people. Groups*,* groups of people like–*,* yeah…If there’s groups of people*,* I feel like they’re looking at me*,* then that tends to be a starting point.*

Interviewer: Yeah, yeah that makes sense, thank you. And you mentioned that you think the psychosis kind of directly caused the depression, and would you say that was related to kind of your thoughts around suicide as well or–,

Participant: Yeah, yeah [Terrie]

A different participant had an active plan to die by suicide because they thought that they, and members of their family, were possessed by the devil. The plan involved drinking spirits and setting fire to their home. They had constant feelings of being persecuted, *“I’ve done something really bad and everyone’s out to get me” [Charlie]* which led to them having suicidal thoughts and active plans involving an overdose of medication. Stress was a strong trigger for their paranoid feelings, thoughts, and beliefs and they described being continually hypervigilant for *“bad things”* that they felt they must have done. Although depressed or low mood states did not seem prominent in their recent suicidal experiences involving stress and paranoia, there was still some indication that their low moods could interact with the emotional toil of living with psychosis:

*And when you’re in psychosis*,* everything’s so negative. Everything is so negative and you have no get up and go and you don’t want to get dressed and you don’t want to tidy the house and you don’t want anyone to face you [Charlie].*

Three aspects of the dynamics comprising these four pathways must be emphasized. First, the pathways did not come across as being entirely linear in nature. Rather, they were intertwined and involved fluctuations and movement between the different psychotic, anxiety-related, and suicidal experiences of participants. Second, memories of previous anxiety episodes or panic attacks could heighten anxiety about having to face those mental health problems again:

*where I know I’ve had that before and I know I’ve had this before*,* and sometimes that puts your fret up a bit ‘cause you don’t want to be in the same instance as a bad experience before [Stevie]*.

Third, and relatedly, across participants, a commonly held view that anxiety abates the more it is experienced was plainly not the case.

## Discussion

The current work used a qualitative approach to better understand the interactions between psychotic, suicidal, and anxiety-related experiences. At the outset, it is important to note that even though some evidence has been collated testifying to the relationships between suicidal thoughts/acts and traditionally conceived psychiatric anxiety disorders [3, 5, 7, 8, 73], relatively minimal research effort has been invested in a comprehensive understanding of the tripartite inter-relationships between psychotic, anxiety-related, and suicidal experiences. Therefore, the current study sought to begin to redress this gap, from which there were three novel findings.

The first novel finding was the emotional and cognitive-emotional effects of the tripartite psychosis-anxiety-suicide interplay which was the inter-linkage between fear, the tangible ‘realness’ of psychotic experiences, and the relentlessness of both psychotic and anxiety-related experiences. Intense fear was often entwined with an over-riding sense that psychotic experiences were real, palpable, and tangible. Fear was amplified when participants did not understand why or how their psychotic and/or anxiety-related experiences were present. A study applying IPA to work with one individual who experienced delusions, also highlighted the concatenations between fear, issues related to control over anxiety, and suicidal thoughts [56]. The impact of fear in relation to suicide has been explored in the context of developing conditions such as acquired immunodeficiency syndrome or cancer, fear of sleep in those dealing with trauma, fear of abandonment, fear of hurting other people, fear of negative evaluations, and fear of relapse in people with schizophrenia [74–79]. Fear is also a discernible component in many forms of social anxiety [80]. However, the confluence of intense fear together with the apparent authenticity, or ‘realness’ of manifestations of paranoia, delusions and hallucinations seemed to give the anxiety-psychosis-suicide dynamic a unique set of characteristics that fed into suicidal states.

Based on a robust and enduring literature [71, 81–86], recent transdiagnostic psychological models of suicide highlight cognitive-emotional perceptions relating to defeat, entrapment, and hopelessness [48, 72, 87]. The second key finding was that patterns capturing the anxiety-psychosis-suicide dynamic in our qualitative data both challenged and extended this focus. The challenge came from perceptions of hopelessness, which although present, were not as prominent as expected. That said, a particularly arresting point made by one of the participants was that it was not so much that they felt hopeless about their future but rather that they felt that they had no future; the future, for them, was absent. In contrast, appraisals of defeat were clearly evident and were in accord with previous conceptualisations and measures of defeat in relation to suicidal experiences [82]. However, the cognitive-emotional components of defeat went beyond, and added to, previous conceptualisations in that feeling both overpowered and overwhelmed were pronounced and escalatory of suicidal thoughts/acts. Similarly, a sense of being ‘thwarted’ was striking. Thoughts and feelings reflecting ‘thwarted belonginess’ are central to the widely-supported Interpersonal-Psychological Theory of Suicide [47] and have been shown to be a strong predictor of suicidal thoughts, plans, and acts across many different clinical settings [88, 89]. ‘Thwarted belonginess’ most obviously applies to social situations in which individuals do not feel connected to others, nor to communities. In the current study, a more fundamental sense of being thwarted was evident which was apparent as a sense of coming to a stop; of not being which was a consequence of the anxiety-psychosis-suicide dynamic.

Similar to hopelessness, perceptions of being trapped also did not arise in a straightforward format from our qualitative data. The kinds of entrapping perceptions that were strongly evident reflected having to live a life of immense, interminable, struggle in which participants felt desperate for their severe mental health problems to end, but that end did not seem possible, other than by suicide. Participants felt trapped because their mental health problems seemed endless and unrelenting. Indeed, permeating the emotional and cognitive-emotional impact of the psychosis-anxiety-suicide dynamic was a powerful impression of the relentlessness of these mental health problems that participants faced. Within the literature, links between suicidal thoughts, plans, and attempts and different manifestations of relentlessness have been documented, such as in the context of chronic pain, degenerative physical conditions, enduring sleep deprivation, unremitting political humiliation, physical and emotional violence, persistent aggression against minority groups, and incessant socio-economic deprivation [90–93]. The impact of perceptions of relentlessness deserves focused scrutiny in relation to both anxiety-related experiences and psychosis, most especially when they interact and are mutually amplifying of suicidal states of mind.

The third key finding emphasised that although the ways in which psychosis, anxiety, and suicidal experiences were highly complex, fluctuating, and dynamic, clear pathways could, nevertheless, be distinguished. In one pathway, anxiety was most proximal to suicidal experiences with psychotic experiences feeding into that anxiety. In a second pathway, psychotic experiences were most proximal to suicidal experiences, with anxiety feeding into those psychotic experiences. These two pathways seemed to be present with delusions, paranoia and/or hallucinations. That said, many participants did not live with just hallucinations or just paranoia/delusions. Rather, they often experienced different modes of hallucinations (e.g., voices, images, smells) together with paranoid and delusional experiences all of which summated and magnified the emotional and cognitive-emotional aspects of psychotic experiences. Developing service-user led measures of the psychological consequences of different modalities of hallucinations [94, 95], and their intersectionality with paranoia and delusions, presents as a necessary future research focus, especially in the context of understanding interactions between anxiety-related and suicidal experiences. One aspect of delineating these pathways should, perhaps, be made clear which is that although explicit or implied mechanisms and pathways were relayed by participants, we did not interpret them to imply cause and effect in the same way that a well-controlled experimental design allows this kind of conclusion. Nevertheless, temporal precedence was indicated (e.g., participants relayed that anxiety seemed to precede paranoia, or that extended anxiety episodes were a precipitant of suicidal thoughts and/or behaviours), as were amplifying effects (e.g., participants stated that anxiety seemed to make paranoia worse), and direct linkages. It is, perhaps, worth noting that in the context of developing and evaluating complex health interventions, a compelling argument has been made for using qualitative work to not only develop and evaluate theoretical mechanisms but to also determine how contextual factors alter their operation [96].The main limitation of the current work was that the qualitative analyses were secondary, meaning that whilst the topic guides deliberately focused on suicidal and psychotic experiences, they were not developed to explicitly explore anxiety-related experiences. This, of course, means that had individuals been asked directly about their anxiety experiences in the context of psychosis and suicide, they may have provided different perspectives and different patterns in the data may have been observed. Nonetheless, this can also be seen as a strength because when participants spoke about anxiety in their interviews, the implication was that these disclosures were spontaneous, participant-led, and therefore, genuinely important to individuals.

A second limitation follows which is that although we explored the influences of anxiety on suicidal experiences with our work with EBEs as we were analysing and interpreting the data, this work was not instantiated at the time of topic guide development. This was because the current work was based on secondary analysis.

A final limitation of this study is that our ability was limited to elicit participants’ perspectives about how wider cultural and societal influences impact their mental health experiences relating to suicide, anxiety, and psychosis. This includes asking participants directly about how other, sometimes entrenched, stereotypical biases may influence the perspectives taken on their mental health experiences, such as their gender identity. We did examine the transcripts for any spontaneous references by participants to the influences of gender stereotyping on the interplay between suicide, psychotic, and anxiety-related experiences, and could find none. Nevertheless, a recommendation for future research is that this issue be explored explicitly and directly with participants, including their perspectives of how stereotypes often inherent in models of health care have affected them. A more general recommendation for future research is to explicitly probe holistic cultural influences on perceptions of mental health problems as personally experienced, in addition to exploring perspectives about cultural influences on communication styles about mental health and provision of mental health resources [97, 98].

Two additional strengths of the current study should be noted. First, participants were deliberately targeted to have both psychotic and acute suicidal experiences. It is relatively rare that the co-occurrence of acute suicidal and psychotic experiences is studied. Second, the analysis was grounded in a systematic use of Framework analysis, a component of which was the development of in-depth summaries for each participant. This grounded both the rigor of the methodology but also the depth in the interpretation.

The cluster of findings identified in the current study present a clear focus for clinical and therapeutic advances which are when working with clients with suicidal and psychotic experiences (i) to explicitly and flexibly explore the role of different manifestations of anxiety in these experiences, including direct and indirect links with suicidal experiences, (ii) to understand the cumulative, relentless, emotional, and cognitive-emotional impact that different forms of psychosis and anxiety can have on a range of suicidal experiences, and (iii) to be unafraid to work with emotions, especially fear.

In conclusion, anxiety and suicide can be directly linked, but with precursors to anxiety from hallucinations, delusions and paranoid thoughts and feelings. Anxiety can also feed-into and amplify psychotic experiences which can, in turn, be directly linked with suicidal experiences. The emotional, and cognitive-emotional effects of living with psychosis have to be better understood in terms of how relentless psychosis together with anxiety can feel; how real psychotic and anxiety-related experiences can seem; and how suicidal states of mind can develop as a desired end, when these kinds of mental health problems appear to have no end [99]. Future directions would benefit from using micro-longitudinal mixed methods designs in conjunction with creative multi-media based qualitative methods [100] to examine the temporal and fluctuating nature of the interplay between psychotic, anxiety-related, and suicidal experiences.

## Supplementary Information

Below is the link to the electronic supplementary material.

Supplementary Material 1

## Data Availability

The raw datasets generated and analysed during the current study are not publicly available as participants did not provide consent for this. De-identified data are available from the corresponding author upon reasonable request.

## Footnotes

1 Glossary of anxiety-related terms available from the corresponding author on request.

## Declarations

### Disclaimers

The CARMS project was funded by the Efficacy and Mechanism Evaluation (EME) Programme, an MRC and NIHR partnership (Reference number: 13/161/25). The views expressed in this publication are those of the author(s) and not necessarily those of the MRC, NIHR or the Department of Health and Social Care. The trial was hosted by Greater Manchester Mental Health NHS Foundation Trust, UK. The Sponsor is Manchester University, UK.
