# Supplementary material for: The dynamic interplay between anxiety-related, psychotic, and suicidal experiences: a qualitative study
Source: BMC Psychiatry. 2025 Nov 19;25:1105. doi: 10.1186/s12888-025-07547-z (PMC12628620; doi:10.1186/s12888-025-07547-z)
Supplement: Supplementary file 1 — Supplementary Material 1 [file 12888_2025_7547_MOESM1_ESM.docx]

**Table S1**

*Examples of suicidal and psychotic experiences as relayed by participants.*

| **Type of experience** | **Indicative quotes** |
| --- | --- |
| Suicidal experiences: Summary Suicidal thoughts, acts, and plans were referred to explicitly by all participants, but could also fluctuate or be less tangible. The function of suicide as an end to intractable and continual mental health problems was patent, but could also be a means to prevent feeling controlled, especially by hallucinations. | |
| Suicide as a state of mind that fluctuated or was in the background. | *“Whereas the suicidal stuff is like it comes and goes, or it's just there in the background” [Sam].* |
| Suicide as an end to suffering and an end to “mental illness”. | *“I think it [suicide] was just not wanting to die, just wanting my illness to end” [Charlie].* |
| Suicide to actualize free will | *“I thought, well, I have to stop giving these beings… the choice to hurt me… It was my free will, you see.” [Tam]*. |
| Psychotic experiences: Summary Participants often had a clear awareness of their hallucinations, paranoia, and delusional thoughts and feelings, and of the impacts of such psychotic experiences on themselves and others. Whilst some participants experienced mainly hallucinations or paranoia, some lived with a number of different types of psychotic experiences (e.g., grandeur and persecution) or lived with both voices and paranoia. It should be noted that not all voices were experienced as negative. | |
| Paranoid feelings, thoughts, and beliefs | *“…paranoia means it’s not based on reality, don’t it?... I’d be paranoid that like, people were out to kill me and stuff like that” [Terrie];*  *“I feel like there’s people waiting outside to get me but when I look – because I can hear them talking about me and stuff – but when I look, they’re not actually there” [Remy]* |
| Paranoia ‘seeded’ by events | *I had a death threat over the phone, I had a death threat through the post and one night when I was walking home, some guy came with a baseball bat and then shot off in a car. So, quite understand, I think most people would start to feel a bit paranoid under, under those circumstance… I had this paranoid seed that was just getting bigger and bigger and bigger…, I kind of er, er, er, erm there was an onset of er delusional psychosis ” [Remy]* |
| Lack of trust | *“It's very difficult because you don't trust — Well, I personally don't trust anybody …Yeah, and everything everyone does and says has a double meaning” [Charlie]*  *“ I just can't trust, I can't even trust my own mind” [Stevie]* |
| Delusional experiences | *“I get a bit delusional.” [Sam]*  *“I can’t describe how horrendous me experiences of this delusional psychosis are but it’s just the closest word that I could put it, it was torture [Morgan]*  *“I also get messages from the TV, music sometimes” [Sam]* |
| Specific Delusions | *“I woke up and realised that I was God, but I'm not…” [Bobbie]*  *“I went into psychosis and I actually planned to drink a bottle of vodka and then set fire to the house, with the children in it, because we were all possessed by the Devil” [Charlie]* |
| Grandiose experiences | *“…when my psychosis first started, I was grandiose, I felt safe! You know, I kinda had these unrealistic delusions that I was a very, very important person in the world, that, that I was best of friends with the prime minister, it was [NAME OF PRIME MINISTER] at the time…” [Morgan]* |
| Premonitions | *“plus I see things before it happens” [Lou]* |
| Visual hallucinations | *“I see things at the corner of the room and feel like they’re dropping acid on me through the ceiling” [Bo]* |
| Auditory Hallucinations/ Voices | *“That voice will like laugh at me when I'm getting stressed. And it will say, ‘Ha ha,’ you know, ‘He's at it again’ and then it will like mock me” [Jude]*  *“I hear voices from people that don’t actually say--, they’re not actually saying anything but I can hear a voice coming from them, stuff like animals as well” [Sam]* |
| Voices as conversations | *“Er, sometimes I try and ignore them and sometimes I talk with them… Just like conversations” [Vic].* |
| Voices as mumbles | *“Yeah sometimes I get voices, it’s like mumbles, yeah” [Ollie]* |
| Many voices | *“I hear about seven different types of voices… but if you can imagine taking your brain out and putting a little person and then they shout” [Georgie]* |
| Voices as a ‘tag-team’ | *“But they're like a trio, yeah, so one gives up. I go, I'm not listening to you, another one will come in, then they'll come in, and then it's replay.” [Rowan].* |
| Voices as a commentary | *“The main one is telling me to hurt myself or to kill myself. But other times they’re just sort of doing like commentary of what I’m doing…in a bad way” [Remy].* |
| Command hallucinations | *"a few voices, telling me to handle a knife, and I ended up picking it up and putting it to my throat, but I didn’t cut myself in the end" [Sam]*  *“Voices tell me to do things, like take overdoses and self-harm” [Kit]* |
| Positive voices | *“my counselling voice”* [Georgie]. |
| Multi-modal hallucinations | *“I have hallucinations of him [a specific god] and dark shadows. Voices tell me to do things, like take overdoses and self-harm” [Kit]* |
| Hallucinations as presences | *“it’s a continual thing of presences or, well I don’t know whether it’s beings or gods or whatever” [Kit]* |
| Hallucinations that also contain delusional content | *“Oh, I see people walking through walls. I see people looking at me. I've seen spaceships. [Short pause]. And I'm convinced they're true” [Robin]* |
| Grandeur and Persecution | *“I have ideas of grandeur and also ideas of persecution” [Charlie]* |
| Dissociative experiences | *“I had one with [name 2], I had a very bad episode of dissociation. And I couldn’t come out of it [Alex]* |
